# Initial Evidence for Symptoms of Postpartum Parent-Infant Relationship Obsessive Compulsive Disorder (PI-ROCD) and Associated Risk for Perturbed Maternal Behavior and Infant Social Disengagement From Mother

**DOI:** 10.3389/fpsyt.2021.589949

**Published:** 2021-09-16

**Authors:** Nathalie Ratzoni, Guy Doron, Tahl I. Frenkel

**Affiliations:** ^1^Ziama Arkin Infancy Institute, Interdisciplinary Center Herzliya, Herzliya, Israel; ^2^Baruch Ivcher School of Psychology, Interdisciplinary Center Herzliya, Herzliya, Israel

**Keywords:** parent-infant relationship, parent-infant relationship obsessive compulsive disorder, perinatal mental health, maternal bonding, maternal behavior

## Abstract

Infant socioemotional development and underlying brain maturation occur primarily within the context of early caregiver-infant relationships. Perinatal research demonstrates detrimental impact of postpartum pathology, including postnatal onset of maternal OCD—on the mother-infant relationship. The present study is the first to examine postnatal onset of a particular dimension of OCD symptoms focusing on close interpersonal relationships (relationship-OCD, i.e., ROCD) within a general population sample. Specifically, we assessed whether symptoms of Parent-Child ROCD (PC-ROCD), may onset postnatally, thus yielding symptoms of Parent-Infant ROCD (PI-ROCD). We adapted the previously validated Parent-Child ROCD measure for use during infancy to assess symptoms of PI-ROCD. The adapted measure, Parent-Infant Relationship Obsessive Compulsive Symptoms Inventory (PI-PROCSI), was administered to 143 mothers from the general population at 4-months postpartum. We investigated concurrent associations between postnatal onset of PI-ROCD, maternal depression and bonding, as well as longitudinal predictive associations with observed maternal and infant behaviors in dyadic interactions at 10 months. Due to dropout across the 1st year postpartum, the subsample with longitudinal data was substantially reduced compared to the full sample. PI-PROCSI scores explained unique variance in concurrent maternal depression over and above concurrent anxiety. PI-PROCSI scores also associated with concurrent impairments of maternal bonding. Moreover, unique associations emerged between maternal PI-ROCD scores and perturbations in both maternal and infant observable behaviors at 10-months. Specifically, observable perturbations in maternal behaviors mediated associations between symptoms of PI-ROCD at 4-months and observable infant avoidance of social engagement behaviors at 10-months. Findings suggest that parent-child ROCD symptoms may onset during the postnatal period, and that such symptoms may play a significant role in shaping quality of reciprocal caregiver-infant interactions. Theoretical and clinical implications are discussed.

## Introduction

Decades of research have established the importance of the early caregiving environment in laying foundations for optimal infant brain development and long-term social and emotional outcomes [for reviews see, (1, 2)]. Specifically, studies reveal that development of infant socioemotional skills and underlying brain maturation occur primarily within the context of the early caregiver-infant relationship, *via* cumulative experience of ongoing reciprocal caregiver-infant interactions [e.g., (3–5)]. As such, factors that interfere with the reciprocal nature of these interactions have attracted a great deal of empirical attention (6–8).

Abundant research reveals remarkable impact of maternal mental health in shaping both maternal and infant behavior within dyadic interactions [for reviews, e.g., (9, 10)]. Perinatal research has demonstrated postnatal onset of various conditions including maternal postpartum depression, anxiety and postpartum Obsessive Compulsive Disorder (OCD). Research documents significant prevalence of these conditions (13–19%, 8.5%, and 9%, respectively), (10–13), their detrimental impact on maternal levels of bonding with her infant [e.g., (14–18)], related substantial perturbations in observed maternal behavior and associated negative infant outcomes [for reviews, e.g., (9, 10, 19–21)].

Recent literature on older children and their parents has identified a specific dimension of parental OCD [partner-focused Relationship Obsessive Compulsive Disorder; (22–24)], that arises within the parent-child relationship and may impact the quality of the emerging parent and infant relationship. Relationship obsessive-compulsive disorder (ROCD) refers to a presentation of OCD focusing on close interpersonal relationships (25). Partner-focused ROCD symptoms is a form of ROCD denoting disabling preoccupation with perceived flaws of the partner (26, 27).

Findings suggest that ROCD symptoms are associated with functional disability, as well as significant personal and relationship distress [e.g., (25, 28, 29)]. Although previous research has established the existence of partner-focused ROCD within the parent-child relationship [e.g., (22–24)], no study has yet assessed whether the onset of parent-child ROCD (PC-ROCD) symptoms may arise during the postnatal period, thus culminating in Parent-infant ROCD (PI-ROCD) symptoms, and whether these might impact the mother and her emerging relationship with her newborn across the 1st month postpartum.

Partner-focused ROCD symptoms include intense preoccupation with the perceived flaws of the partner [e.g., (27, 30)]. Such preoccupations may center on a wide range of domains including physical features (e.g., nose, body-proportions), social qualities (e.g., social skills, humor), competence (e.g., being successful), and personality attributes such as morality, intelligence or emotional stability (24, 31). Partner-focused ROCD symptoms are also characterized by various compulsive behaviors including repeated checking (e.g., of the partner's behaviors or competencies), comparisons (e.g., between the partner's characteristics and those of others), neutralizing (e.g., visualizing positive situations in the relationship), and reassurance seeking (e.g., “I often seek reassurance from friends, family, etc. about whether my partner is smart enough”) (32).

Within the parent-child context, ROCD symptoms are characterized by parental preoccupation with the child's perceived flaws (23, 24). In a recent online study, for instance, 1.2% of 350 parents of children between the ages of 12–18 recruited from the general community in the US, reported spending above 3 h a day being preoccupied with the flaws in their eldest child's appearance, personality or aptitude. Further, 0.6% of parents reported that such preoccupation significantly interfered with their functioning, and 0.6% reported substantial associated distress (23).

Parents may experience unwanted intrusive thoughts, images or urges pertaining to their child's perceived flaws (e.g., memory of a specific instance where the child “failed”). Such intrusions trigger fears of future harm occurring to the child (e.g., s/he will be bullied in school) or distress of the mere occurrence of the thought (e.g., “I'm a bad parent for dwelling on this”). As PC-ROCD intrusions often contradict parental values (e.g., “All children should be accepted no matter their flaws”) they may also be associated with parental feelings of guilt and shame.

As in other types of OCD, PC-ROCD intrusions often provoke compulsive behaviors in order to alleviate the distress caused by the content or occurrence of the unwanted intrusion (22). PC-ROCD symptoms compulsions include repeated comparisons of the child qualities, behaviors or character to other children (including siblings), checking of the child's behaviors, and reassurance seeking regarding the child's perceived flaws or incompetency.

Parent-child ROCD symptoms in non-clinical samples have been associated with parental self-vulnerabilities (e.g., parental self-contingencies on specific domains such as intelligence and appearance) and over-reliance of parental self-worth on the child perceived value (24). ROCD symptoms focusing on the child's perceived flaws have also been strongly associated with parental distress and negative experience of parenting (23). Such symptoms have been associated with increased parental stress, as well as parental depression and anxiety over and above other parental OCD symptoms (23). Given the negative impact of parent-child ROCD symptoms focusing on the child's flaws on parental well-being, it was suggested that such symptoms may be disruptive to both the quality of parent caregiving and to the quality of the parent-child relationship (23).

Indeed, research on children of parents with other types of OCD, reveal that these children often experience difficulties within the relationship with the symptomatic parent (33, 34). Compared to healthy controls, mothers with OCD were found to express less warmth toward their children, show more criticism, and promote less psychological autonomy as observed in interactions with their child at the ages of 7–14 years (33). At 6 months postpartum, mothers with OCD were less sensitive to their infants (20). Furthermore, at the ages of 7–18 years, parental OCD symptoms appears to increase risk for child psychopathology—including both symptoms of depression and anxiety (35). One would expect to find similar and perhaps greater negative relational impacts in the specific context of PC-ROCD.

### The Present Study

Consistent with previous findings suggesting OCD symptoms may onset during the postnatal period [(20, 36); for review see (37)], the objectives of the present study were 2-fold. Firstly, we examined whether PC-ROCD symptoms may onset within the general population during the postnatal period, thereby yielding symptoms of Parent-Infant ROCD (PI-ROCD).

Although PC-ROCD symptoms have been suggested to occur early in the parent child relationship (23), no research has examined potential onset of these symptoms within the general population as early as infancy. To this end, we adapted the previously validated parent-child Partner-focused Relationship Obsessive Compulsive Symptoms Inventory [PROCSI-PC; (23)], for use in the parent-infant context (PI-PROCSI). We employed the items that comprise the previously validated PROCSI-PC questionnaire, in addition to 3 additional items which we hypothesized would be particularly relevant for parenting during infancy. To attain our specific study aims, and in line with previous reports which demonstrated ROCD symptoms in non-clinical populations (24, 31), we administered the adapted Parent-Infant Partner-focused Relationship Obsessive Compulsive Symptoms Inventory (PI-PROCSI) in a general sample of mothers at 4-months postpartum, and employed a sample size sufficient for conducting reliable Factor Analysis (38, 39). We employed exploratory factor analysis (EFA) and confirmatory factor analysis (CFA) to examine the factor structure of the ROCD measure which was adapted for use in the parent-infant context.

Second, we examined the potential impacts of PI-ROCD symptoms on mother, infant, and the emerging relationship between the two. Specifically, given the focus of PI-ROCD on perceived flaws of the infant, we sought to examine whether increased symptoms of PI-ROCD might associate with impaired maternal bonding toward her infant (i.e., mother's warm and positive emotions and thoughts toward her infant). Maternal bonding has been shown to be an important precursor of supportive parenting (40, 41).

Furthermore, we sought to examine whether symptoms of PI-ROCD measured at 4-months postpartum, would translate into observable perturbations in maternal behavior within dyadic interactions with her infant at the age of 10-months. Specifically, given maternal preoccupation with infant flaws, and compulsive seeking of reassurance regarding perceived flaws and incompetency—we expected PI-ROCD symptoms to associate with increased maternal expression of criticism (or decreased praising) within dyadic interactions.

Finally, we expected maternal criticism to elicit infant avoidance of social engagement with mother thereby exerting detrimental impact on the ongoing reciprocal nature of dyadic interactions. Moreover, we sought to assess whether perturbations in maternal behaviors (in the form of impaired praising or increased criticism), would mediate predictive associations between symptoms of PI-ROCD at 4-months and infant avoidance of social engagement with mother at 10-months. Initial evidence for a longitudinal pathway of risk within the general population, from early postnatal symptoms of PI-ROCD to perturbed infant behavior toward the end of the 1st year of life, would underscore the relevance of the identified factors and emphasize the need for future research aimed at replicating and extending the present report to complete validation of an early screening tool in a large-scale heterogenous sample comprised of both non-clinical and clinical populations. The mediating role of maternal behavior, would inform preventive interventions, suggesting that detrimental impact of PI-ROCD symptoms on infant social engagement with mother may be ameliorated by targeting maternal behavior—thus further underscoring the potential benefit of future validation of early screening tools.

Postpartum symptoms of depression and anxiety tend to co-occur with OCD [e.g., (42, 43)], and have been found to exert detrimental impacts on the emerging mother and infant relationship [for reviews see e.g., (9, 21)]. Thus, in the present study, we sought to assess the unique potentially detrimental effect of PI-ROCD symptoms on both mother and infant above and beyond comorbid postnatal symptoms of maternal depression and anxiety.

## Materials and Methods

### Participants and Procedure

The current sample was comprised of a general population sample of 143 Israeli women recruited during the third trimester of pregnancy as part of a broader longitudinal study.

Data were collected at two study timepoints. At 4-months postpartum (Infant Mean age = 4.34 months, SD = 0.55), participants were visited in their homes. Self-report questionnaires assessed maternal level of depression and anxiety [ASR; (44)], parent-infant ROCD symptoms (PI-PROCSI), as well as levels of maternal subjective bonding with the baby [PBQ; (45)]. At 10-months postpartum (Infant mean age = 9.91 months, SD = 1.21), a 5-min mother-infant freeplay interaction was videorecorded during a laboratory visit for the assessment of maternal and infant behaviors (i.e. maternal level of praising/criticism toward her infant, and level of infant social disengagement from mother).

Out of all participants, one had missing data for maternal depression and anxiety, 13 had missing data for the maternal bonding measure and 45 have missing data for observed maternal and infant behaviors, due to dropout. At time of recruitment, mothers were aged between 20 and 44 years (*M* = 31.71, *SD* = 3.93); had between 10 and 21 years of education (*M* = 15.56, *SD* = 2.30). The majority of mothers (81.4%) were born in Israel; had between 1 and 4 children (*M* = 1.62, *SD* = 0.80); 51.7% male. The study was approved by the author's Institutional Review Board (IRB) and all participants provided their written consent.

### Measures

#### PI-ROCD Symptoms 4-Months Postpartum

The parent-infant version of the PROCSI (PI-PROCSI) is an adapted version of the previously validated Parent-Child-Related Obsessive-Compulsive Symptoms Inventory [PC-PROCSI; (23)]. The adapted measure is comprised of the PROCSI-PC items which were re-worded to refer to “infant” instead of “child.” In addition, three items were added to assess sleeping and eating patterns (e.g., “I seek reassurance from friends, family, etc. about whether my baby's sleeping/eating patterns match his age”) and general development (e.g., “I keep looking for evidence that my baby's development in various fields is normal”). The resulting 32 item Parent-infant Partner-focused Related Obsessive-Compulsive Symptoms Inventory (PI-PROCSI) was administered to participants. Participants rated the extent to which such thoughts/behaviors describe their feelings on a scale ranging from 0 “not at all” to 4 “very much”). In line with taxometric studies of OCD indicating that symptoms are better conceptualized as continuous (46), Continuous ROCD scores were calculated, with higher scores indicating more symptoms.

#### Maternal Levels of Depression and Anxiety 4-Months Postpartum

Maternal postpartum depression and anxiety were assessed using the Achenbach Adult Self-Report (ASR) of the Achenbach System of Empirically Based Assessment (ASEBA) (44). Comprised of 126 items, mothers rated their emotional/social/behavioral problems, on a 3-point Likert scale. We calculated T-scores of the DSM-oriented Depression and Anxiety scales. Higher scores indicate more symptoms.

#### Maternal Bonding With Her Infant at 4-Months Postpartum

Maternal bonding with her infant was assessed using the Postpartum Bonding Questionnaire [PBQ; (45)] which detects relationship disturbances as expressed by maternal hostility, aggression, lack of emotion, and rejection toward her infant. The original questionnaire consists of 25 statements rated on a 6-point scale with higher scores indicating healthier bonding. Two items were omitted from the current study because of their low reliability reported in the literature (47). Total scores were calculated as an average of all remaining 23 items.

#### Observed Maternal Behavior at 10-Months Postpartum

Level of maternal expression of praising/criticism toward her infant was coded offline by two experienced coders from a 5-min video-recorded freeplay interaction collected during a laboratory visit at the age of 10 months. Mothers and infants were seated on a playmat with a fixed set of age-appropriate toys. Mothers were instructed to “play with her infant as she normally does.” The researcher waited in a control room. Three synchronized video cameras were used to videorecord the freeplay interactions allowing for detailed and accurate coding of maternal and infant behavior offline, using the Maternal Praising Scale of the Coding Interactive Behavior [CIB; (48)] coding scheme. This 5-point Likert scale refers to the extent to which mother provides verbal praising to infant's behavior, when appropriate, for example when infant achieves a goal or makes an effort.

#### Observed Infant Social Engagement With Mother at 10-Month Postpartum

Level of infant social engagement with mother was coded offline by two experienced coders from the same 5-min freeplay interaction described above. Infant social engagement was indexed using the following 5-point Likert scales of the Coding Interactive Behavior [CIB; (48)] coding scheme: “Infant social initiation” (i.e., the extent to which the infant initiates a social bid toward mother; “Infant gaze toward mother” (i.e., the extent to which infant gaze turned toward mother or toward an object in joint attention with mother); “Infant vocalization” (i.e., all positive vocalizations directed toward mother); “Infant positive affect” (i.e., infant expression of laughs, smiles, and vocalizations indicating positive engagement with mother). A mean composite score of all four scales was calculated, due to significant positive correlations between the four (all *r*'s > 0.41, all *p*'s < 0.01).

A subsample of comprising of 20% of the videos were double coded for calculation of inter-rater reliability yielding 90.9% agreement between raters for maternal praising and 87.42% for the infant social engagement composite. Kappa tests for each of the observed variables indicated high reliability as follows: maternal Praising = 0.860 *p* < 0.001, Infant social initiation = 0.820 *p* < 0.001, Infant Gaze toward mother = 0.939 *p* < 0.001, infant Vocalization = 0.538 *p* < 0.001, and Infant Positive affect = 0.757 *p* < 0.001.

### Data Analytic Strategy

In order to test the factor structure of the items of the PI-PROCSI we conducted an exploratory factor analysis (EFA) with Promax rotation, in SPSS followed by a confirmatory factor analysis (CFA; AMOS, version 25.0). For the EFA, Items that showed low loading (<0.4) or cross-loading (<0.2) were removed from analysis. Based on criteria of eigenvalue>1 and scree plots, the number of factors was selected and labeled by content. First-order and second-order theory-based models were then entered separately into a confirmatory factor analysis (CFA; AMOS, version 25.0). Goodness of model fit was determined based on the comparative fit index (CFI), the root-mean-square error of approximation (RMSEA) and the standardized root mean square residual (SRMR).

Next, pearson correlations were conducted between all study variables, including demographics, followed by a series of hierarchical regressions. Bonferroni corrections were performed to address issues of multiple comparisons. First, we examined the predictive role of the PI-PROCSI factors in predicting concurrent depression, above and beyond anxiety. Second, we examined the predictive role of the PI-PROCSI factors in predicting concurrent anxiety, beyond depression. Lastly, we examine the predictive value of the PI-PROCSI factors in predicting concurrent bonding, beyond depression and anxiety. Control variables were entered in the first step while PI-PROCSI factors were entered in the second step.

Finally, we tested the indirect effects separately for each of the PI-PROCSI factors to explore whether maternal praising behavior at 10 months mediated the link between PI-PROCSI at 4 months and infant social engagement at 10 months. A 95% bootstrap confidence interval (CI) were obtained for the indirect effects. The bootstrap CI was generated by using the percentile bootstrap estimation method and 2,000 bootstrap samples. An index of mediation that is different from zero (i.e., 95% bootstrap CI does not include zero) indicates the significance and strength of the indirect effect.

## Results

### PI-PROCSI Factor Structure and Psychometric Properties

As expected in a general population sample, initial scanning of the PI-PROCSI data revealed a positively skewed distribution. A square root transformation was therefore performed. All 32 items were included in an exploratory factor analysis (EFA) with Promax rotation. Ten items showed low loading or cross-loading and were thus removed from analysis. Based on criterion of eigenvalue > 1 and scree plot, the remaining 22-items were grouped into eight factors, which explained 68.40% of the total variance. All items loaded above 0.63 on their primary factor; none of the secondary loading exceeded 0.28. One of the factors was comprised on a single item and was therefore removed.

The seven factors of the 21-item version of the PI-PROCSI were labeled based on the focus of their obsessive-compulsive symptoms. Five factors referred to maternal obsessive preoccupations with infant's current state: (1) compulsions related to development; (2) distress from obsessions related to development; (3) parental perception of infant's physical appearance; (4) the extent to which parent perceives infant's sleeping and eating patterns as “normal;” (5) the extent to which parent feels an urge to “compare” her infant to other babies.

**Figure 1:**
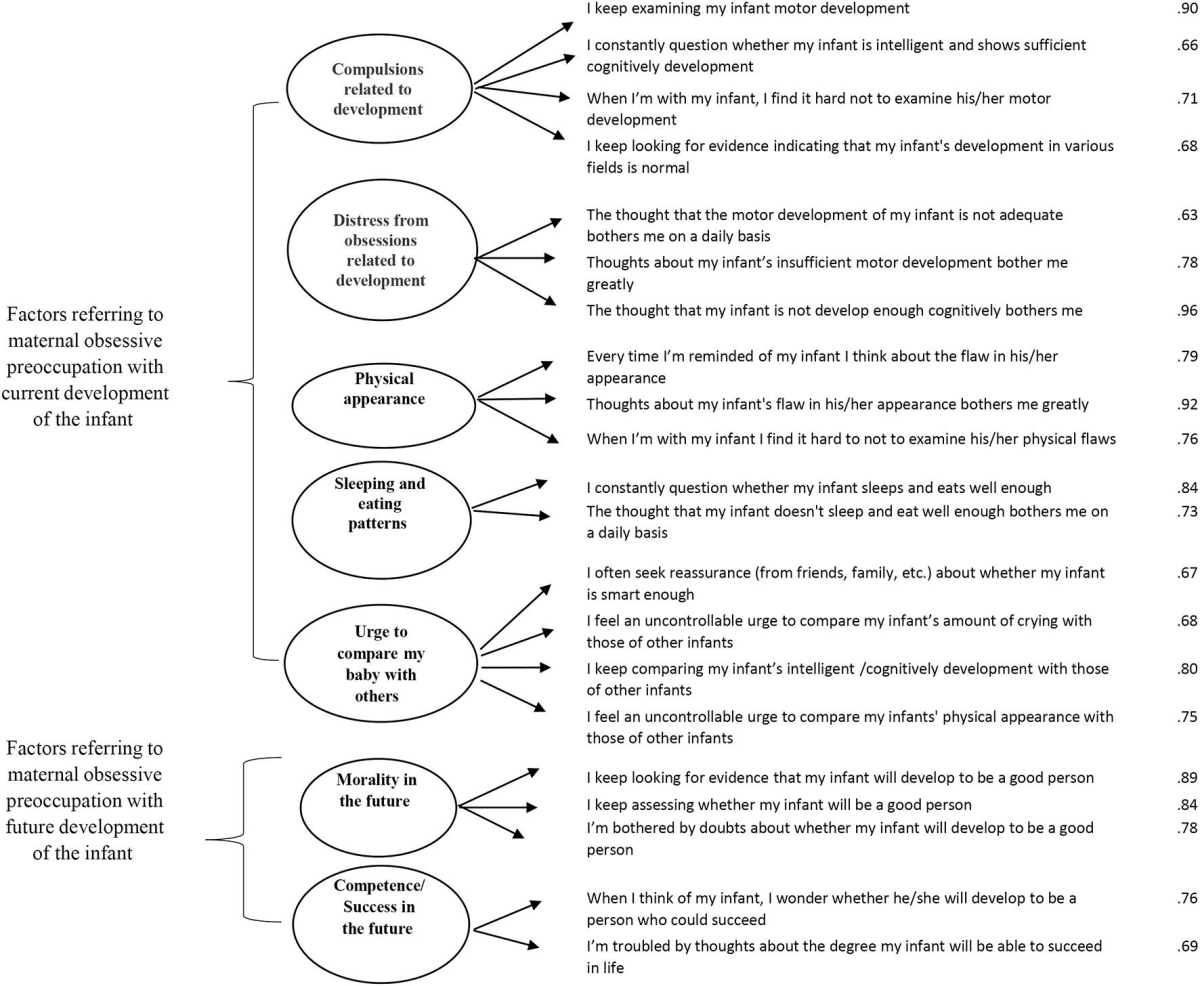
Confirmatory factor analysis model of PI-PROCSI. Error variance and covariance are omitted. Factor loadings were obtained using principal components extraction with promax rotation (*N* = 143).

The two remaining factors referred to parental preoccupation with infants' future development: (6) the extent to which infant would develop into a moral person and (7) the extent to which infant would be competent/successful in the future (see Figure 1 for factors and loadings).

We used confirmatory factor analysis (CFA; AMOS version 25.0) to test model fit of the EFA factor structure. CFA results were evaluated using the χ^2^ statistic the root mean square error of approximation (RMSEA), the standardized root mean square residual (SRMR) and comparative fit index (CFI). The seven factor model showed good fit (CFI = 0.95, RMSEA = 0.04, SRMR = 0.06).

Since parents tend to be preoccupied with their infants' current and future development, we also tested a two-factor second-order structure including obsessive preoccupation regarding current infant development and those regarding his/her future (PI-PROCSI current and future development, see Figure 1). This model showed acceptable fit (CFI = 0.94, RMSEA = 0.05, SRMR = 0.07) with adequate reliability scores for both second-order factors (Cronbach alpha = 0.88 and 0.81 for the PI-PROCSI current and future development factors, respectively).

The final scale comprised of 21 items that can be coded either as a seven-factor scale or a two-factor scale. The more specific seven-factor coding might be useful for clinical applications, whereas the two-factor coding might be useful for empirical investigations, and was used in the current study (PI-PROCSI Current development Mean = 0.73, SD = 0.55, Median = 0.63, Range = 3; PI-PROCSI Future development Mean = 0.67, SD = 0.71, Median = 0.40, Range = 3.6).

### Correlations of PI-PROCSI Symptom Scores at 4-Months With Maternal Demographics, Concurrent Symptoms of Depression and Anxiety, Concurrent Maternal Bonding, and Observed Maternal and Infant Behaviors at 10 Months

Pearson correlations were calculated between all variables (see Table 1). PI-PROCSI “current” and “future” factors (i.e., symptoms referring to preoccupation with current and future development of the infant) were positively related to concurrent symptoms of maternal depression and anxiety. PI-PROCSI current and future factors were also negatively related to concurrent maternal bonding revealing links between maternal symptoms of PI-ROCD and impaired bonding toward her infant.

**Table 1:** Correlations matrix between demographic variables, PI-ROCD (PI-PROCSI current and future development), maternal depression and anxiety (ASR), maternal bonding (PBQ), and maternal praising and infant social engagement (CIB).

	**1**	**2**	**3**	**4**	**5**	**6**	**7**	**8**	**9**	**10**	**11**
1. Maternal age	–										
2. Maternal education	0.24^**^	–									
3. Family income	0.29^**^	0.39^**^	–								
4. Number of children in the family	0.22^**^	0.21^*^	0.15	–							
5. PI-PROCSI current dev (4 mos)	0.12	0.01	0.12	−0.06	–						
6. PI-PROCSI future dev (4 mos)	0.05	−0.12	−0.07	0.04	0.57^**^	–					
7. Maternal depression (ASR, 4 mos)	0.05	−0.03	0.05	0.07	0.41^**^	0.24^**^	–				
8. Maternal anxiety (ASR, 4 mos)	0.02	0.01	0.08	0.07	0.40^**^	0.28^**^	0.65^**^	–			
9. Maternal bonding (PBQ, 4 mos)	−0.06	−0.07	−0.09	0.00	−0.45^**^	−0.24^**^	−0.51^**^	−0.46^**^	–		
10. Maternal praising (CIB, 10-mos)	−0.02	0.17	−0.05	0.05	−0.02	−0.27^**^	0.19	0.10	−0.14	–	
11. Infant social engagement (CIB, 10-mos)	−0.01	0.25^**^	0.09	0.09	0.02	−0.28^**^	0.06	−0.04	−0.07	0.42^**^	–

* *Correlation is significant at the 0.05 level.*

** *Correlation is significant at the 0.01 level*.

In addition, symptoms of PI-PROCSI future (i.e., preoccupation referring to future development of the infant), were longitudinally predictive of both maternal behavior and infant social disengagement from mother. Specifically, maternal preoccupation with future development of her infant at 4 months, predicted decreased expression of maternal praising toward her infant at 10-months as well as decreased infant social engagement with mother.

### Incremental Predictive Value of the PI-PROCSI Symptom Scores in Predicting Concurrent Symptoms of Anxiety and Depression

Hierarchical regression was performed to assess the incremental predictive value of the PI-PROCSI- current and future development scores in predicting concurrent parental depression over and above maternal anxiety. Symptoms of PI-PROCSI current development significantly predict concurrent depression over and-above symptoms of PI- PROCSI future development and maternal anxiety (*R*^2^ change = 0.03, *p* < 0.016). Symptoms of PI-PROCSI future development, did not predict concurrent depression, beyond symptoms of PI-ROCD current development and maternal anxiety (*R*^2^ change = 0.00, ns). Similarly, symptoms of PI-PROCSI current and future development did not significantly predict concurrent anxiety over and-above maternal depression at 4-months (*R*^2^ change = 0.00, ns for both PI-ROCD current and future).

### Concurrent Associations Between PI-PROCSI Symptom Scores and Maternal Bonding at 4-Months

Hierarchical regression analysis examined associations between PI-PROCSI symptoms and concurrent maternal bonding over and above concurrent symptoms of maternal depression and anxiety. Results showed that higher symptoms of PI-PROCSI current development were significantly negatively associated with maternal bonding (*R*^2^ change = 0.05, *p* < 0.016, see Table 2). PI-PROCSI future development scores, however, did not contribute significantly to the model (*R*^2^ change= 0.00, ns).

**Table 2:** Regression of maternal bonding on PI-PROCSI current and future development, maternal depression and anxiety (*N* = 129).

		**Maternal bonding (PBQ)**		
			**Confidence interval (b)**	
	**B**	**β**	**Lo95**	**Up95**
Maternal depression (ASR)	−0.03	−0.32^**^	−0.05	−0.01
Maternal anxiety (ASR)	−0.01	−0.13	−0.03	0.01
PI-PROCSI current dev	−0.26	−0.27^**^	−0.45	−0.07
PI-PROCSI future dev	0.02	0.03	−0.11	0.15

** *Correlation is significant at the 0.01 level (2-tailed)*.

### Associations Between PI-PROCSI Symptoms at 4-Months and Infant Social Engagement Behaviors at 10-Months: the Mediating Role of Maternal Praising Behaviors at 10-Months

**Figure 2:**
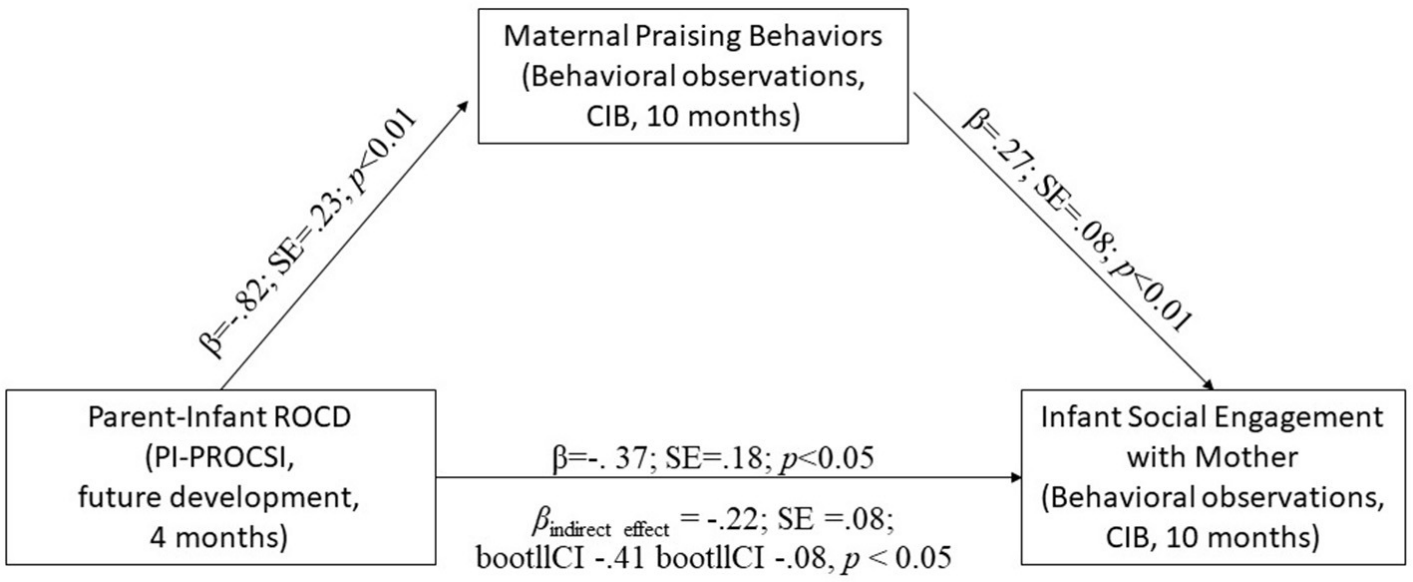
Standardized regression coefficients between PI-PROCSI future development at 4 months and infant social engagement at 10-months mediated by maternal praising at 10-months, controlling for maternal depression and anxiety at 4-months (*N* = 97).

Mediation analyses yielded a significant indirect link between PI-PROCSI future development symptom scores at 4-months and decreased infant social engagement behaviors at 10-months. Specifically, higher symptoms of PI-PROCSI future development were associated with lower levels of maternal praising at 10-months that, in turn, predicted higher levels of infant social engagement behaviors at 10-months, above and beyond maternal depression and anxiety (β_indirecteffect_ = −0.22; SE = 0.08; bootllCI−0.41 bootllCI−0.08, *p* < 0.016; see Figure 2). No mediation effects were found for maternal praising in mediating associations between symptoms of PI-PROCSI current development at 4-months and infant behaviors at 10-months (β_indirecteffect_ = −0.19; SE = 0.13; bootllCI−0.51 bootllCI.01, ns).

## Discussion

Decades of research findings have established that reciprocal caregiver-infant interactions play a key role in socioemotional development and underlying maturation of the infant brain [for reviews see, (1, 2)]. As such, research on perinatal maternal mental health has drawn substantial empirical attention demonstrating the potential interference of maternal postpartum pathology on the reciprocal nature of these interactions [for reviews, e.g., (9, 10)]. The particular detrimental and long-lasting effects of postpartum depression, for instance, have been well-documented—yielding impressive public awareness and assimilation of preventive policy within perinatal health care systems [e.g., (49)]. Comparatively less attention has been paid to postnatal anxiety symptoms, and even less to the specific risk for postnatal onset of OCD symptoms.

While previous literature has evidenced postnatal onset of OCD symptoms [(20, 36) for review see (37)], the present study is the first to examine postnatal onset of a particular dimension of OCD symptoms focusing on interpersonal relationships—relationship OCD (ROCD), within the general population. Indeed, close to a decade of research has linked ROCD symptoms with significant disability and interference in romantic [e.g., (25–27, 29, 31, 50)] and parent-child relationships (23, 24). The present study, therefore, investigated the associations between postnatal onset of ROCD symptoms and caregiver-infant interactions.

In order to do this, we first adapted the previously validated measure of Parent-Child ROCD [PROCSI-PC; (23)], for use in the parent-infant context (Partner-Infant Relationship Obsessive Compulsive Symptoms Inventory; PI-PROCSI). The adapted measure was found to be internally consistent, and factor analysis indicated two global factors or seven more content specific factors. Evaluating the incremental predictive value of PI-PROCSI symptomology, we found that PI-PROCSI symptom scores explained unique variance in concurrent maternal depression over and above concurrent maternal anxiety. Our results also revealed significant links between PI-PROCSI scores, concurrent impairments of maternal bonding and predictive associations with observable perturbations in both maternal and infant behaviors within dyadic interactions. The latter observable, longitudinal, effects of parent-infant ROCD symptoms on maternal and infant behaviors held above and beyond those of maternal depression and anxiety. Taken together, the present report provides initial evidence for postnatal onset of PC-ROCD symptoms and identifies factors that should be included in future validation of a PI-ROCD screening measure. Initial evidence for predictive value of the identified factors suggest that the PI-PROCSI may capture a distinct theoretical construct which may play an important role in the shaping of reciprocal caregiver-infant interactions, thus underscoring the need for future validation of the PI-ROCD screening measure.

Moreover, the findings from our study delineate a mediating mechanism for the longitudinal pathway of risk through which parent-infant ROCD symptoms might interfere with the ongoing reciprocal nature of interactions between mother and her infant. Specifically, our results indicate that perturbations in maternal behaviors (in the form of impaired praising or increased criticism), mediate predictive associations between symptoms of PI-ROCD at 4 months and infant avoidance of social engagement with mother at 10-months. These findings emphasize the need for early screening and inform the planning of preventive interventions, suggesting that targeting maternal behaviors may effectively moderate risk.

This present study had a number of limitations. First, we lack the assessment of fathers and were thus unable to examine the father's role in the interplay between maternal ROCD symptoms and the mother-infant relationship. Second, we assess symptoms of ROCD only across the first 4 months postpartum. Repeated measurement of PI-ROCD across the entire postpartum period is necessary to delineate the exact onset and time course of symptomology. Third, the present study employed a relatively small homogeneous non-clinical sample, in which symptom scores were positively skewed, variability was small and the majority of subjects reported a very low symptom severity. Noteworthy, the use of non-clinical populations within OCD research in general (51), and ROCD research in particular, is a common practice (24, 27). Previous OCD literature reveals substantial impairments in non-clinical populations [e.g., (52)], and previous ROCD literature reveals that ROCD symptoms are associated with OC related beliefs in both clinical and non-clinical samples (24, 27, 31). Accordingly, taxometric studies of OCD have found that OCD symptoms and OC related beliefs are better conceptualized as continuous and dimensional rather than categorical (46). Indeed, the present study examined potential obsessive compulsive symptoms in the general population, but not a clinical diagnosis of a specific type of OCD. The present non-clinical sample displayed significant associations between ROCD symptoms and both maternal and infant behaviors. These findings suggest that non-clinical levels of ROCD symptomology may warrant substantial preventive efforts—as these may exert meaningful negative effects on the emerging relationship between a mother and her infant. Nonetheless, individuals with clinical diagnosis of ROCD may differ from non-clinical participants in symptom severity and the degree of impairment (24). Future large scale validation in a heterogenous sample is necessary for the identification of clinical cutoff levels and prevalence of PI-ROCD diagnosis, as well further research within clinical samples as well.

Finally, while some suggest that the currently employed sample size is sufficient for conducting reliable factor analysis in line with the present preliminary study aim (38, 39, 53), it is generally accepted that a larger sample size would be required to increase stability of our result (54). Thus, complete validation of the PI-PROCSI based on the identified factors, requires future replication and extension in a largescale heterogenous sample comprised of both non-clinical and clinical populations (55). A larger sample is further warranted given the relatively high dropout rate evidenced in the present study. Literature reviewing perinatal research indicate comparable dropout rates ranging from 20 to 35% in non-clinical samples [e.g., (56)], and dropout rates appear to be particularly high in studies employing observational methods [e.g., (57)]. While dropouts did not differ from remaining subjects, with respect to anxiety, depression or bonding, they did however appear to have higher levels of PI-ROCD symptoms for future development relative to non-dropouts. This finding may indicate that mothers with relatively higher symptom scores of ROCD had more difficulty to continue study participation. The fact that the reported effects were found despite the dropout of mother's displaying relatively higher levels of symptomology, might suggest that effects may be stronger than presently demonstrated. Future replication studies need to further examine this possibility.

Taking these limitations into account, the current study has important theoretical and clinical implications pointing toward a novel avenue of research for perinatal mental health. Specifically, the results of our study implicate PI-ROCD symptoms in the caregiver-infant relationship. Specifically, preoccupation with the infant's future morality and competence may reduce praising, increase parental criticism leading to infant's avoidance of social engagement. This, in turn, may further increase parental fears and preoccupation with the child's morality and competence reinforcing a vicious cycle. Identifying and targeting parental fears and preoccupations with the future development of the child may promote healthier caregiver-child interaction.

Noteworthy, maternal preoccupations and concerns are extremely common during the postpartum period (often termed “primary maternal preoccupation”). Previous literature indeed reveals some resemblance in both content and character between typical maternal concerns and OCD symptoms during the postpartum period (58–60). Critically though, while typical maternal preoccupation generally exerts positive effects on maternal behavior (e.g., heightened sensitivity to infant cues, feelings of intense love and idealization of the infant), and preoccupation fade gradually without treatment in the first few months, OCD symptoms have been found to have negative effects for the infant and if left untreated, may have a long-term course and effect (61). In line with this, the finding of significant associations between ROCD symptoms and perturbations in both maternal and infant behavior—appear to suggest that ROCD symptoms measured in the present study index more than just typical postpartum preoccupation. Future research is necessary to directly assess links between ROCD and typical postpartum maternal preoccupation. Given the natural tendency of mothers to idealize their infant during the early postpartum period (61), even minor levels obsessive thoughts regarding infant flaws during the early postpartum period may exert particularly amplified effects.

Furthermore, there is likely high comorbidity between PI-ROCD symptoms and additional anxiety symptoms typical in the postpartum period. To this end, the current study explored ROCD symptoms, above and beyond concurrent maternal postpartum depression and anxiety. Findings reveal that ROCD symptoms uniquely contribute to maternal and infant behavior—above and beyond more general postpartum anxiety.

Finally, our findings also involve parent-infant ROCD symptoms in the development of post-natal depression. Indeed, previous research has shown parent-child ROCD symptom predict depression symptoms over and above other parental OCD symptoms (23). High comorbidity between postnatal depression and anxiety, have also brought authors to suggest that anxiety symptoms may play a particularly strong role in the etiology of postpartum depression [e.g., (43)]. Future research is necessary to assess whether the co-occurrence of PI-ROCD may potentially confer a worse prognosis than postpartum depression alone.

## Data Availability Statement

The raw data supporting the conclusions of this article will be made available by the authors, without undue reservation.

## Ethics Statement

The studies involving human participants were reviewed and approved by Interdisciplinary Center (IDC) Herzliya, Israel. Written informed consent to participate in this study was provided by the participants' legal guardian/next of kin.

## Author Contributions

TF is principle investigator at the site of data collection in the Ziama Arkin Infancy Institute. TF, GD, and NR developed the study concept and study design which comprised part of NR's Master's thesis under the supervision of TF and GD. Testing and data collection were performed by NR. NR performed the data analysis, interpretation under the supervision of TF and GD, and drafted the manuscript under the supervision of TF and GD. All authors approved the final version of the manuscript for submission.

## Funding

Funding for this study was provided by the Arkin Family donation to the Ziama Arkin Infancy Institute, IDC, Herzliya.

## Conflict of Interest

The authors declare that the research was conducted in the absence of any commercial or financial relationships that could be construed as a potential conflict of interest.

## Publisher's Note

All claims expressed in this article are solely those of the authors and do not necessarily represent those of their affiliated organizations, or those of the publisher, the editors and the reviewers. Any product that may be evaluated in this article, or claim that may be made by its manufacturer, is not guaranteed or endorsed by the publisher.
